# Lack of *in vitro* effect of aglepristone on IFN-γ and IL-4 production by resting and mitogen-activated T cells of luteal bitches

**DOI:** 10.1186/1746-6148-9-220

**Published:** 2013-10-26

**Authors:** Piotr Jurka, Lidia Szulc-Dąbrowska, Joanna Borkowska, Anna Winnicka

**Affiliations:** 1 Department of Small Animal Diseases with Clinic, Laboratory of Small Animal Reproduction, Warsaw, Poland; 2 Department of Preclinical Sciences, Division of Immunology, Warsaw, Poland; 3 Department of Pathology and Veterinary Diagnostics, Division of Animal Pathophysiology, Faculty of Veterinary Medicine, Warsaw University of Life Sciences-SGGW, Nowoursynowska 159c, 02-767 Warsaw, Poland

**Keywords:** Aglepristone, Mifepristone, Bitch, T cells, Cytokines

## Abstract

**Background:**

Aglepristone (RU534) is an antiprogestin used for pregnancy termination, parturition induction and conservative pyometra treatment in bitches. Its molecular structure is similar to mifepristone, an antiprogestin used in human medicine. Mifepristone has been shown to suppress proliferation and cytokine production by T cells, whereas the effect of aglepristone on T cell function remains elusive. The purpose of this project was to investigate the *in vitro* influence of RU534 on IFN-γ and IL-4 synthesis by peripheral blood T cells isolated from healthy bitches (N = 16) in luteal phase. The peripheral blood mononuclear cells (PBMCs) were incubated with three different dosages of aglepristone, or dimethyl sulfoxide (DMSO), with or without mitogen. The production of cytokines by resting or mitogen-activated T cells was determined by intercellular staining and flow cytometry analysis or ELISA assay, respectively.

**Results:**

Our results showed no statistically significant differences in the percentage of IFN-γ and IL-4-synthesizing CD4^+^ or CD8^+^ resting T cells between untreated and aglepristone-treated cells at 24 and 48 hours post treatment. Moreover, mitogen-activated PBMCs treated with RU534 displayed similar concentration of IFN-γ and IL-4 in culture supernatants to those observed in mitogen-activated DMSO-treated PBMCs. Presented results indicate that administration of aglepristone for 48 hours has no influence on IFN-γ and IL-4 synthesis by resting and mitogen-activated T cells isolated from diestral bitches.

**Conclusions:**

We conclude that antiprogestins may differentially affect T cell function depending on the animal species in which they are applied.

## Background

Aglepristone (RU534) is the only registered antiprogestin in veterinary medicine. This nuclear progesterone receptor (nPR) antagonist has a higher affinity for nPR than progesterone itself [1]. This medication was initially used for pregnancy termination. Currently, aglepristone is used for induction of parturition and in the treatment of canine and feline pyometra [2]. Recently, there were also attempts to use aglepristone in the therapy of progesterone-dependent disorders in dogs, such as: growth hormone excess [3], vaginal fibroma [4] and mammary gland carcinoma [5].

Aglepristone is known to block the progesterone action on target cell receptors. In bitches aglepristone abolishes the inhibitory effect of progesterone on the uterine myometrium. Studies conducted *in vitro* have demonstrated that aglepristone enhances contractile response of myometrial fibers to oxytocin and prostaglandin PGF2alpha during metestrus [6]. The administration of aglepristone during the early luteal phase in healthy non-pregnant bitches shortened the interestrous interval suggesting that aglepristone influences the hypothalamic-pituitary-ovarian axis [7].

Aglepristone is a very effective drug in conservative treatment of canine pyometra. It is thought that pyometra is linked to a hormonal imbalance and progesterone dominance in luteal phase which, in turn, suppresses the local innate immunity and favours bacterial colonization [8]. Since progesterone probably plays a major role in the pathogenesis of pyometra, pharmacological blockade of nPR by aglepristone may lead to fast recovery [9]. *In vivo* studies have shown that bitches with pyometra 14 days post treatment with aglepristone showed a decreased number of monocytes and granulocytes compared to reference values [10]. Furthermore, studies by Fieni and collogues [11] have indicated that inhibition of nPR by aglepristone in bitches with pyometra significantly reduced the leukocyte count and plasma progesterone concentrations over the course of treatment. After 48 hours of aglepristone administration bitches with closed pyometra showed cervical opening with subsequent evacuation of purulent discharge from uterus and improvement in the animal’s condition [11]. However, the exact mechanism of aglepristone action in the treatment of pyometra remains unknown. We can only suppose that aglepristone may have an influence on reversion of immune suppression induced by progesterone.

Much of our current understanding of the potential effect of aglepristone on canine immune cells comes from studies of the mifepristone (RU486), the first synthesized antiprogestin used in human medicine. Mifepristone is now classified as a selective progesterone-receptor modulator (SPRM) due to its mixed antagonist/agonist action on PR. Additionally, it is an antagonist/agonist of the glucocorticoid receptor (GR) [12]. Mifepristone has a very similar molecular structure to aglepristone [1]. In humans mifepristone is used for early termination of pregnancy and in the therapy of progesterone-dependent tumors [13]. Mifepristone was successfully used for pregnancy termination in dogs [14]. It also exerts an anti-glucocorticoid effect in this species. In dogs RU486 alters adrenal function by inducing an increase in plasma adrenocorticotropic hormone (ACTH) and cortisol concentrations [15,16].

It has been demonstrated that mifepristone suppressed proliferation and downregulated the interleukin-2 receptor (IL-2R) mRNA in human lymphocytes. Moreover, mifepristone acted as a GR agonist and inhibited secretion of IL-2 and IL-3 by phytohemagglutinin (PHA)-activated normal human peripheral blood lymphocytes (NPBL) [17]. Mifepriston enhanced cytotoxicity of peripheral blood NK cells isolated from woman in implantation phase [18] and uterine NK (uNK) cells isolated at the window of implantation [19]. Additionally, RU-486 inhibited suppressive effect of P4 on IFN-γ mRNA expression in uNK cells stimulated with CpG and IL-12. The same effect was observed in murine splenic NK cells isolated in diestrus [20].

Bitches in luteal phase are under immunosuppression. PBMCs isolated form bitches in diestrus showed decreased proliferation in response to lipopolysaccharide (LPS) derived from *E. coli* and PHA compared to cells isolated in other phases of estrus cycle [21,22]. Data concerning pyometra treatment and mifepristone action suggest that aglepristone may have an influence on canine immune cells.

For that reason, the aim of the present study was to investigate the *in vitro* effect of aglepristone on cytokine synthesis by resting and mitogen-activated T cells isolated from bitches in luteal phase.

## Methods

### Animals

In the study 16 healthy bitches at different age (9 months - 7 years, average 2 years) and different breeds were used. All bitches were in luteal phase (2 weeks after estrus) confirmed by anamnesis, clinical examination, cytology and peripheral blood progesterone concentration assay. Investigations were carried out after the obtaining an agreement from III-rd Local Animal Experimentation Committee at the Warsaw University of Life Sciences number lke 72/2009 and an agreement of the Dean of the Faculty of Veterinary Medicine, Warsaw University of Life Sciences number 1/2009. Such permissions are necessary before the receipt of the grant from the National Science Centre, Poland and are in accordance with the Law of United Europe.

### Blood samples and hormonal profiles

Blood samples were collected from the supraradial vein every 7 days starting from the 1st day after estrus (confirmed by hormonal examination and vaginal cytology). Blood for *in vitro* investigation was taken on day 14 of the luteal phase. Measurements of progesterone concentrations were performed using commercial immunoenzymatic tests for quantitative determination of P4. Measurement of optical density was taken with a Pointe 2000 apparatus (Pointe Scientific, Poland). Each analysis was performed two times in each series. Efficiency of extraction oscillated between 92% and 99%. Assay sensitivity and intra-series errors were 0.05 ng/ml (0.8 nmol/l) and 8.0% respectively for P4.

### Isolation of PBMCs

Before isolation of PBMCs, EDTA peripheral blood was centrifuged at 250 × *g* for 20 min to obtain plasma which was collected and stored on ice until use. PBMCs were isolated from the remaining EDTA peripheral blood diluted twofold with two-times concentrated RPMI 1640 medium (Gibco, USA) using 50% (v/v) Percoll (Sigma–Aldrich) gradient with centrifugation at 400 × *g* for 30 min. Collected PBMCs were washed twice with RMPI 1640 and red blood cells were then lysed in NH_4_Cl/Tris buffer for 10 min on ice. After washing, PBMCs were resuspended in culture medium RMPI 1640 supplemented with 10% (v/v) fetal bovine serum (FBS, HyClone, USA) and 1% (v/v) antibiotic–antimycotic (100 U/ml penicillin, 100 μg/ml streptomycin, 250 ng/ml amfotericin) (Sigma–Aldrich) at 5 × 10^6^/ml.

### PBMCs treatment and stimulation

For enumeration of cytokine-synthesizing T cells by flow cytometry, PBMCs were placed at 5 × 10^5^/well in a flat-bottom 96-well plate and treated with increased concentrations of aglepristone (Alizine, Virbac, France) diluted in dimethyl sulfoxide (DMSO) i.e. 30, 300 and 3000 ng or with DMSO alone, as a negative control. After 24 and 48 h of treatment, PBMCs were restimulated using 50 ng/ml of phorbol 12-myristate 13-acetate (PMA, Sigma–Aldrich) and 4 μg/ml of ionomycin (Sigma–Aldrich) in the presence of 15 μg/ml brefeldin A (BD Biosciences, USA) for 4 h at 37°C in 5% CO_2_[23].

For measurement of cytokine concentration by ELISA, PBMCs seeded in a flat-bottom 96-well plate were treated with DMSO or 30, 300 and 3000 ng of aglepristone together with mitogens: concanavalin A (Con A, 1 μg/ml; Sigma-Aldrich) or PHA (10 μg/ml; Sigma-Aldrich). After 24 and 48 h supernatants were collected and stored at -20°C for further experiments.

### Surface and intracellular staining

After restimulation PBMCs were washed in phosphate buffered saline (PBS) and blocked for nonspecific binding in 30% (v/v) FBS in PBS for 30 min on ice. Surface staining was performed using monoclonal antibodies (mAb) conjugated with fluorescein isothiocyanate (FITC) against canine CD4 or canine CD8 (both from AbD Serotec, UK) surface molecules. Intracellular staining was then performed using Cytoix/Cytoperm kit (BD Biosciences) in accordance with the manufacturer’s instructions. Briefly, cells were fixed and permeabilized with Cytoix/Cytoperm solution for 25 min on ice followed by washing in Perm/Wash solution. Next, cells were stained for 30 min on ice with the respective phycoerythrin (PE)-labeled mAb directed against bovine IFN-γ or bovine IL-4 (both from AbD Serotec). Cross-reactivity of these mAbs with canine cytokines was previously described [24]. After washing, PBMCs were fixed in 2% paraformaldehyde (PFA, Sigma–Aldrich) in PBS and analyzed by flow cytometry within 24 h. In all experiments negative controls were run with each set of samples.

### Flow cytometry analysis

Analysis of intracellular cytokine staining was performed using FACSCalibur flow cytometer equipped with the CellQuest software (Becton Dickinson, USA). Results were determined as a percentage of CD4^+^ or CD8^+^ T cells synthesizing cytokine, and as mean fluorescent intensity (MFI) of a cytokine-positive CD4^+^ or CD8^+^ T cell subpopulation. Data from 2 × 10^4^ events were acquired for each sample. Lymphocytes were gated by the region determined by forward (FSC) and side (SSC) scatter characteristics.

### ELISA assay

ELISA for canine IFN-γ and IL-4 was performed using kits purchased from R&D Systems (USA) and EIAab (China), respectively, according to the manufacturer’s instructions. Kits had a detection sensitivity of 60 pg/ml and 7, 8 pg/ml for IFN-γ and IL-4, respectively.

### Data analysis and statistics

The percentage of cytokine-producing T cells and the MFI of cytokine-positive T cells-treated with aglepristone were presented relative to control values. The concentrations of cytokines in culture supernatants of mitogen-stimulated PDMC-treated with aglepristone were also presented relative to control values. All data were expressed as mean ± standard deviation (SD). Differences between aglepristone-treated and untreated control PBMCs at indicated time points were calculated using the Student’s t-test for paired samples (in case of normal data distribution) or nonparametric Wilcoxon signed rank test (STATISTICA 6.0 software, StatSoft). Statistical significance was determined at P ≤ 0.05.

## Results

### Synthesis of IFN-γ and IL-4 by CD4^+^ and CD8^+^ T cells-treated with aglepristone

We examined the *in vitro* effect of aglepristone on the synthesis of IFN-γ and IL-4 by canine CD4^+^ and CD8^+^ T cells isolated from peripheral blood of bitches in the luteal phase. The concentration of progesterone in blood of the studied bitches was high and ranged from 25,3 ng/ml to 36,7 ng/ml (fairly 30,9 ± 6,3 ng/ml). To achieve synthesis of cytokines, T cells were restimulated *in vitro* with ionomycin and PMA for 4 h, which was chosen to be an optimal time for restimulation (data not shown). For all the experiments, the percentage of cytokine-producing T cells and MFI of cytokine-positive T cells treated with aglepristone were normalized to the DMSO-treated control cells. At first, we assessed the *in vitro* influence of aglepristone on the percentage of T cell subsets (CD4^+^ and CD8^+^). Our results demonstrated no effect of aglepristone on the CD4^+^/CD8^+^ T cells ratio (data not shown) after 24 and 48 h of treatment.

**Figure 1:**
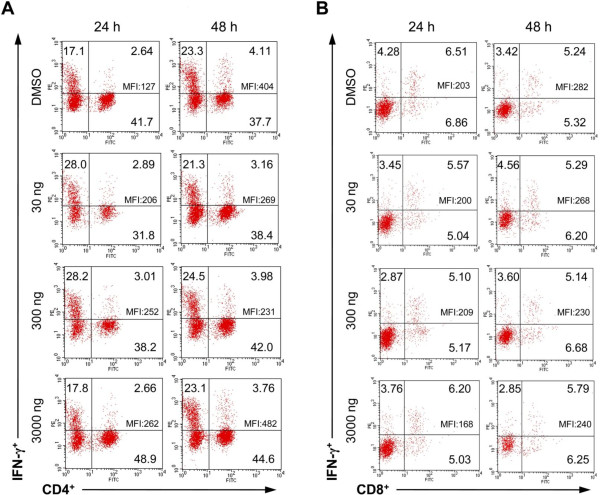
**Representative dot plots show synthesis of IFN-γ by CD4**^**+ **^**(A) and CD8**^**+ **^**(B) T cells isolated from diestral bitches and treated for 24 and 48 h with DMSO (control) or 30, 300 and 3000 ng/ml of aglepristone.** Numbers within quadrants represent the percentage of positive cells for a given marker within the gate for lymphocytes. MFI of IFN-γ in T cell subsets is shown within upper right quadrants.

**Figure 2:**
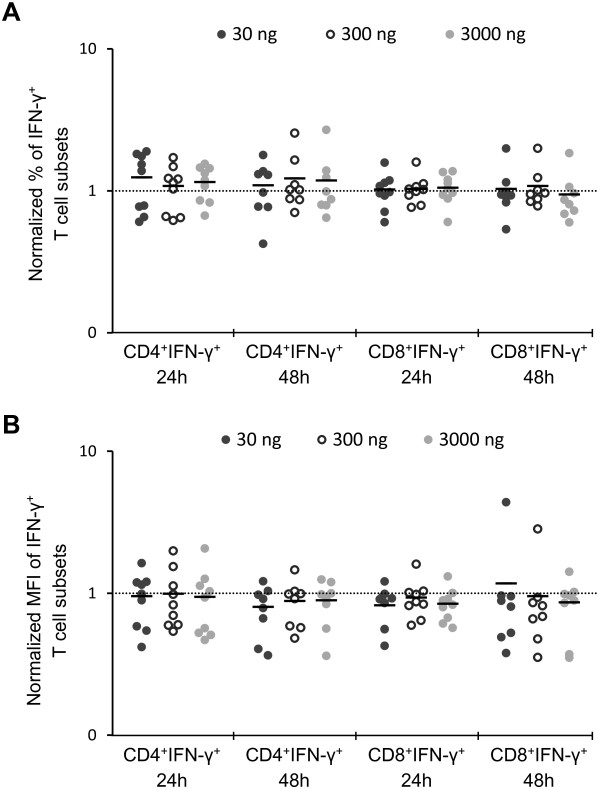
**The percentage of IFN-γ-synthesizing T cell subsets (A) and the MFI of IFN-γ-positive T cell subsets (B) treated for 24 and 48 h with 30, 300 and 3000 ng/ml of aglepristone.** All data are presented relative to control values (line). Each point represents an individual dog (n = 7–9), with the bar indicating the mean. *P < 0.05, ** P < 0.01.

Representative dot blots showing synthesis of IFN-γ by CD4^+^ and CD8^+^ T cells isolated from diestral bitches and *in vitro* treated with DMSO (control) or 30, 300 and 3000 ng/ml of aglepristone, are presented on Figure 1. The percentage of CD4^+^ T cells-synthesizing IFN-γ ranged from 6.0% to 18.1% and 4.7% to 18.6% in control cells, 5.2% to 27.7% and 4.2% to 24.0% in cells treated with 30 ng/ml of aglepristone, 4.3% to 22.1% and 6.9% to 20.5% in cells treated with 300 ng/ml of aglepristone, 5.2% to 26.3% and 4.1% to 20.2% in cells treated with 3000 ng/ml of aglepristone, at 24 and 48 h of incubation, respectively. Our results showed no differences in the percentage of CD4^+^ T cells-synthesizing IFN-γ between control and studied groups (Figure 2A). Moreover, flow cytometric analysis revealed no effect of aglepristone on MFI of IFN-γ produced by CD4^+^ T cells (Figure 2B). The percentage of CD8^+^ T cells-synthesizing IFN-γ in control and aglepristone-treated groups ranged from 15% to around 60% at 24 and 48 h of incubation. Similarly to CD4^+^ T cells, CD8^+^ T cells synthesized IFN-γ at similar levels in the control and aglepristone-treated groups (Figure 2).

**Figure 3:**
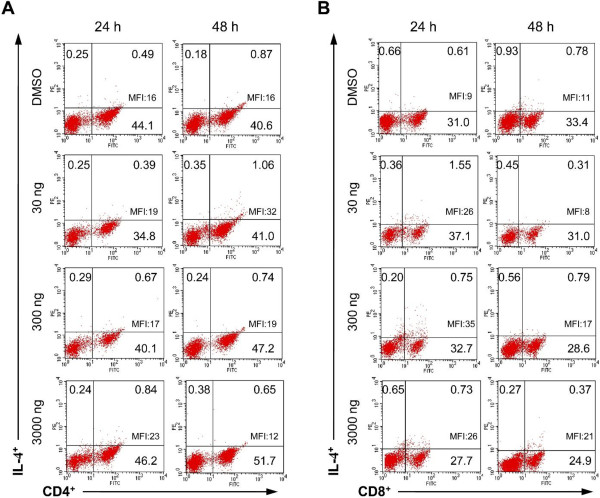
**Representative dot plots show synthesis of IL-4 by CD4**^**+ **^**(A) and CD8**^**+ **^**(B) T cells isolated form bitches in luteal phase and treated for 24 and 48 h with DMSO (control) or 30, 300 and 3000 ng/ml of aglepristone.** Numbers within quadrants represent the percentage of positive cells for a given marker within the gate for lymphocytes. MFI of IL-4 in T cell subsets is shown within upper right quadrants.

**Figure 4:**
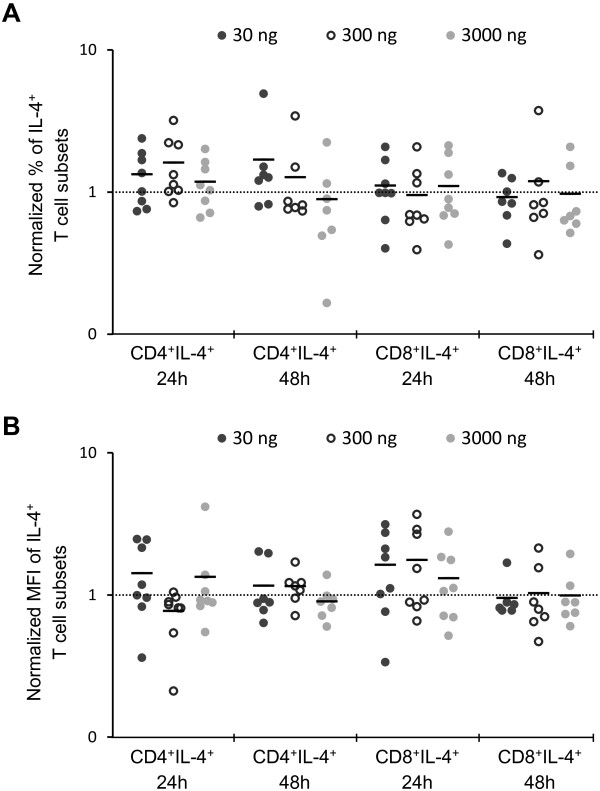
**The percentage of IL-4-synthesizing T cell subsets (A) and the MFI of IL-4-positive T cell subsets (B) treated for 24 and 48 h with 30, 300 and 3000 ng/ml of aglepristone.** All data are presented relative to control values (line). Each point represents an individual dog (N = 7–8), with the bar indicating the mean. *P < 0.05, ** P < 0.01.

The percentage of both CD4^+^ and CD8^+^ T cells-synthesizing IL-4 ranged from around 1% to around 4% in control group and groups treated with different dosages of aglepristone (Figure 3). Our results indicated no statistically significant changes in the percentage of IL-4-synthesizing CD4^+^ and CD8^+^ T cells in group treated with aglepristone compared to control group (Figure 4A). Moreover, in both T cell subsets no statistically significant changes in MFI of IL-4 expression were observed between control and studied groups (Figure 4B).

### No effect of aglepristone on IFN-γ and IL-4 production by mitogen-activated PBMCs

**Figure 5:**
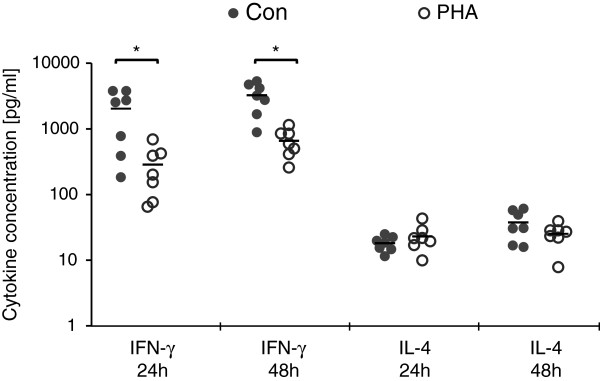
**The production of IFN-γ and IL-4 by ConA- or PHA-activated control PBMCs at 24 and 48 h of culture.** Each point represents an individual dog (N = 7), with the bar indicating the mean. *P < 0.05, ** P < 0.01.

Next, we examined whether aglepristone influences the production of cytokines by mitogen-activated PBMCs. Because ConA and PHA was shown to stimulate T but not B cells [25], we can assume that most IFN-γ and IL-4 in culture supernatant derived from activated T cells. PBMCs stimulation with ConA or PHA significantly increased IFN-γ and IL-4 production compared to unstimulated cells (data not presented). Moreover, ConA-stimulated PBMC produced significantly (P ≤ 0.01) more IFN-γ than PHA-stimulated cells (Figure 5), however the proliferation activity was higher in the latter cells (data not presented).

**Figure 6:**
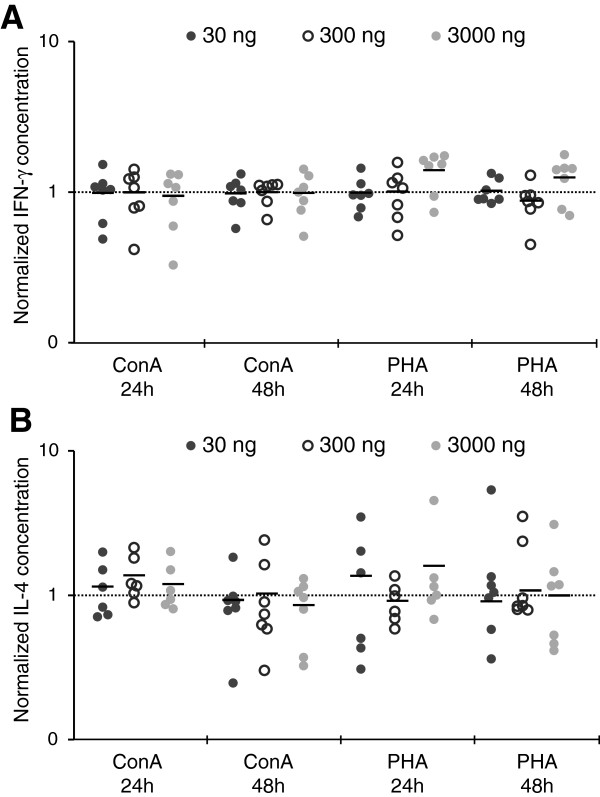
**The normalized concentration of IFN-γ (A) and IL-4 (B) produced by PBMNs after 24 and 48h of treatment with 30, 300 and 3000 ng/ml of aglepristone.** All data are presented relative to control values (line). Each point represents an individual dog (N = 7–8), with the bar indicating the mean. *P < 0.05, ** P < 0.01.

Our results showed that aglepristone has no effect on IFN-γ and IL-4 production by canine PBMCs, induced neither by ConA nor PHA (Figure 6). The level of IFN-γ and IL-4 in culture supernatants of aglepristone-treated cells was comparable with control cells, treated only with DMSO.

## Discussion

This is the first study on the *in vitro* influence of aglepristone on IFN-γ and IL-4 synthesis by resting CD4^+^ and CD8^+^ T lymphocytes and mitogen activated PBMCs isolated from luteal bitches. The concentration of P4 in the luteal phase of bitches is always very high and suppresses the function of the immune system cells [2].

The results of our study showed that there are no statistically significant differences in the percentage of CD4^+^ and CD8^+^ T cells-synthesizing IFN-γ or IL-4 between the control group and groups treated with different dosages of aglepristone. Moreover, we did not observe the dose-effect relationship between aglepristone exposure and the production of cytokines by T cell subsets. Based on our results we can conclude that aglepristone has no *in vitro* effect on resting CD4^+^ and CD8^+^ T cell differentiation toward T helper 1 (Th1) or Th2 and T cytotoxic 1 (Tc1) or Tc2 phenotype, respectively.

The second analyzed parameter was MFI of cytokines synthesized by T lymphocyte subsets. Statistical analysis did not reveal any significant differences in IFN-γ or IL-4 synthesis by resting Th1 or Tc1 and Th2 or Tc2 cells, respectively, treated with aglepristone compared to untreated control cells. Moreover, aglepristone did not suppress mitogen-derived production of IFN-γ and IL-4 by PBMCs isolated from bitches in luteal phase. Our results indicate that aglepristone has no suppressive effect on IFN-γ and IL-4 synthesis by resting and mitogen-activated T cells.

It is known that mifepristone, an analogue of aglepristone, inhibits many human T cell function. Mifepristone alone or synergistically with progesterone was shown to inhibit a PHA-induced proliferation of human T cells [26]. It also suppressed a production of IL-3 and IL-2 by those cells [17]. It is thought that its suppressive effect on human T cell function is mediated by agonistic action on GR [27]. Moreover, mifepristone suppressed in a dose-dependent manner a rapid non-genomic responses caused by progesterone, such as increased intracellular calcium (Ca^2+^) and decreased intracellular pH in human T cells [26]. No effect of aglepristone on the synthesis of IFN-γ and IL-4 may suggest that aglepristone at a concentration < 3 μg/ml does not affect the activation of canine T lymphocytes. The maximal concentration of aglepristone after 2 injections of 10 mg/kg/day at a 24 h interval is achieved after 2.5 days and reached 280 ng/ml. It cannot be excluded that aglepristone has no influence on T cell cytokine synthesis in *in vivo* conditions. Moreover, it cannot be excluded that aglepristone performs a modulatory effect on T cells activation/function at a higher concentration (> 3 μg/ml). Mifepristone was shown to have a significant dose dependent inhibitory influence on proliferation of PHA-stimulated human T cells at 5 μM and above [26]. The effect of mifepristone on canine T cell function has not been investigated to-date, thus it is not known whether a potential mechanism of inhibition of T lymphocyte properties by RU486 is similar in human and canine T cells. Additionally, we cannot exclude that aglepristone is not an agonist of canine GR or has low affinity to GR. However, it is still possible that aglepristone affects the production of other cytokines by T cells and/or their proliferation. These matters need further examination.

We also have to remember about potential differences between aglepristone and mifepristone action. Mifepristone is a selective receptor modulator and its action on target cells has not been clearly defined. It shows mixed agonist/antagonist affinity to both GR and PR. The type of mifepristone action depends on the cell type [28], receptors level [29], promoter context [30] and level of modulatory molecules in cell environment [28]. The agonistic action of mifepristone on GR has been studied in different cells lines transfected with pMTVCAT, where acetyltransferase (CAT) gene was under regulation of glucocorticoid-inducible promoter. The agonist activity of mifepristone depended on cell line and GR expression. Expression of glucocorticoid related genes after mifepristone treatment increased proportionally to GR level in those cells. Agonistic effect of mifepristone was noticeable only in cells with higher GR expression [29]. Liu and coworkers [28] demonstrated that mifepristone has different influence on transcription activity on P4 related genes in different cell types. It correlates with the ratio of endogenous coactivators/corepressors in those cells [28]. The aglepristone action on canine T cells is hard to define based on mifepristone data, because of many factors involved in its interaction with target cells. For that reason, future studies are needed to determine factors influencing the aglepristone action on target cells including affinity to GR, promoter context, type of cells and presence of modulatory molecules in the cellular environment.

The exact mechanism of aglepristone action is not known. There are two types of antiprogestins. Type II refers to molecules that bind to the receptor and promote its binding to HREs (hormone response elements) of target genes. Binding of antiprogestin-receptor complex to HREs does not activate transcription, because of inefficient conformation charges of the receptor [29]. Type I antiprogestins bind to the receptor but do not promote its binding to DNA. This mechanism of action is limited to passive blockage of the receptor. Mifepristone is type II antiprogestin [31]. In contrast to mifepristone, onapristone – type I antiprogestin, has no influence on transcription of progesterone related genes. Onapristone is considered to be pure antagonist of PR. It shows only minimal affinity to GR [12], however *in vivo* studies in rats may suggest its potential agonistic and antagonistic action on GR [32].

It is not known whether aglepristone is a type I or type II antiprogestin. Onapristone differs from mifepristone not only in C17 chain structure but also in stereochemical conformation at C18 [33]. It is thought that the affinity to GR is related to the bend structure at A/B ring junction of steroid skeleton [34]. Aglepristone has the same stereochemical structure as mifepristone. The only dissimilarity between these two compounds is C17 structure. Aglepristone has 1-propenyl side chain at the 17α position, whereas mifepristone has a 17α-1-propinyl group [1]. We cannot exclude that even slight differences in this chain structure may impact the conformation of the whole molecule. The molecule conformation is directly correlated with its affinity to the receptors and mechanism of action. It seems that even a slight change in ligand structure affects the coactivator interaction with nuclear receptors [35]. Additionally, in different animal species receptors vary in terms of structure. For example, structure of conservative amino acid motif in canine nPR vary from that in human nPR [36]. Mifepristone also shows different affinity to receptors depending on the species [1].

## Conclusions

In summary, our results demonstrated no effect of aglepristone on the synthesis of IFN-γ and IL-4 by resting or mitogen-activated T cells of peripheral blood of canine bitches in the luteal phase. Future studies are clearly needed to evaluate the influence of aglepristone on proliferation of mitogen-activated canine T cells. Obtained results will help to understand the immunomodulatory effect of antiprogestins on functional properties of canine immune cells.

## Competing interests

None of the authors have any conflict of interest to declare.

## Authors’ contributions

PJ drafted the manuscript. LSD, PJ, JB provided data and managed the data records. JB performed statistical analyses. LSD, PJ, JB, AW reviewed and commented the manuscript during its preparation. All authors read and approved the final manuscript.
